# Studies on the antibacterial activity of the antimicrobial peptide Mastoparan X against methicillin-resistant *Staphylococcus aureus*

**DOI:** 10.3389/fcimb.2025.1552872

**Published:** 2025-05-29

**Authors:** Zhangping Lu, Xiaofang Liang, Wenbo Deng, Qianqian Liu, Yulin Wang, Meng Liu, Fugui Lin, Zhihong Liu, Yu Zhang, Wenjie Wang, Yingying Sun, Yaozhou Wu, Lianhua Wei

**Affiliations:** Department of Clinical Laboratory, Gansu Provincial Hospital, Lanzhou, Gansu, China

**Keywords:** Mastoparan X, MRSA, USA 300, antimicrobial peptide, antimicrobial activity, biofilm

## Abstract

**Background:**

Methicillin-resistant *Staphylococcus aureus* (MRSA) poses a serious threat to the public health system due to its multi-drug resistance and strong biofilm-forming ability. Here, we explored the possible inhibitory mechanism of an antimicrobial peptide, Mastoparan X, against MRSA

**Methods:**

Minimum Inhibitory Concentration (MIC) and Minimum Bactericidal Concentration (MBC) of Mastoparan X against MRSA USA300 were determined by microbroth dilution method. The antibacterial activity of Mastoparan X against USA300 was then evaluated by time-growth curves, membrane fluidity, reactive oxygen species(ROS), flow cytometry, scanning electron microscopy (SEM) and confocal laser scanning microscopy (CLSM). In addition, the inhibitory and scavenging effects of Mastoparan X on USA300 biofilm were evaluated using crystal violet staining. Finally, gene expression changes in USA300 after treatment with Mastoparan X were analyzed by transcriptomics and verified by RT-qPCR.

**Results:**

The MIC and MBC of Mastoparan X on USA300 were 32 μg/mL and 64 μg/mL, respectively. SEM observation showed significant changes in cell morphology after Mastoparan X treatment. Flow cytometry confirmed that Mastoparan X promoted the apoptosis of MRSA cells. In addition, Mastoparan X inhibited the formation of MRSA biofilm while destroying the mature bioepithelia already formed. Transcriptomic analysis showed that 851 genes were significantly altered and ABC transport protein, amino acid biosynthesis, glycolysis and tricarboxylic acid (TCA) cycle were inhibited after 16μg/mL Mastoparan X treatment.

**Conclusion:**

Our study demonstrated that Mastoparan X has potent bactericidal activity against MRSA and is expected to provide new potential peptides for the clinical treatment of MRSA.

## Introduction

*Staphylococcus aureus* (*S. aureus*) is one of the most common pathogens in community- and hospital-acquired infections. Although *S. aureus* is known to be part of the normal skin microbiota, it is commonly the cause of infections, including skin, soft tissue, blood, and respiratory tract infections (Lee et al., 2018). The problem of antimicrobial drug resistance has been exacerbated by antibiotic misuse and lack of regulation, with the emergence and rapid spread of drug-resistant bacteria and drug-resistant genes leading to the development of resistance in *S. aureus* to almost all available antibiotics (Costa et al., 2024). MRSA will remain a “priority antibiotic-resistant pathogen” in the World Health Organization’s updated list of continuing antibiotic-resistant pathogens in 2024 for research and development of new antimicrobial drugs (World Health Organization (WHO), 2024). Currently, vancomycin and linezolid are among the few antimicrobial agents used against MRSA infections. However, strains with moderate resistance to vancomycin and linezolid have recently emerged globally (Kato et al., 2021). In addition, MRSA biofilm-associated infections are more challenging to treat with antibiotics due to their potent biofilm-forming ability (Guo et al., 2022). This is important because *S. aureus* can be 100 to thousands more resistant to common antibiotics within biofilms. Thus, MRSA biofilms are a significant contributor to treatment failure, persistent infections, increased risk of medical device replacement, and increased morbidity and mortality in most MRSA-associated infectious diseases (Wang et al., 2020). Developing new antimicrobial drugs is time-consuming and costly, leading many pharmaceutical companies to opt-out (Koh Jing Jie et al., 2022).If this trend continues, it will threaten the prevention and treatment of antimicrobial infections. Given this, the urgent development of new antimicrobial drugs for treating MRSA-induced infections is a matter of urgency. Given the growing challenges posed by *S. aureus*’s versatile pathogenicity and toxin-mediated virulence mechanisms, particularly in the era of antimicrobial resistance, the exploration of alternative therapeutic strategies like antimicrobial peptides (AMPs) has become imperative to address these multifaceted threats.

Antimicrobial peptides (AMPs) are a class of small molecule peptides produced by the body’s innate immune system that can penetrate bacterial cell membranes and transport proteins. They are now considered to be the best alternative to antibiotics (Li et al., 2022; Luo and Song, 2021). Mastoparan X (MPX), an antimicrobial peptide extracted from the venom sacs of swarming wasps, contains acidic and basic residues (including three basic residues) with a net charge of 4 and exhibits good antimicrobial activity against Gram-positive (*Staphylococcus aureus* ATCC 25923), Gram-negative pathogens (*Escherichia coli* ATCC 25922; Salmonella enterica serovar Typhimurium CVCC 541), and the opportunistic fungal pathogen *Candida albicans* ATCC 90029 (Zhao et al., 2021). It was shown that modulating the hydrophobicity of peptides by introducing unnatural amino acids with octyl side chains through amino acid substitutions at positions 1, 8, and 14 enhances the bactericidal capacity of the antimicrobial peptide MPX (Henriksen et al., 2014). However, the antimicrobial activity of MPX against MRSA and the inhibitory mechanism remains unclear. Therefore, in this study, we aimed to explore the *in vitro* antibacterial activity of MPX against MRSA and its underlying mechanism.We planned to evaluate the inhibitory effect of MPX by determining the MIC and MBC, as well as conducting time-growth curve analysis. We also intended to use flow cytometry, scanning electron microscopy, and CLSM to elucidate the impact of MPX on the membrane integrity and apoptosis of MRSA cells. Additionally, we aimed to investigate the influence of MPX on the formation and disruption of MRSA biofilms. Finally, transcriptomic analysis will be performed on MRSA treated with sub-inhibitory concentrations of MPX to identify potential changes in gene expression and pathway inhibitions. MPX has antimicrobial activity against MRSA, laying the foundation for developing alternatives to conventional antibiotics.

## Materials and methods

### Bacterial strains and growth conditions

The MRSA USA300 strain was procured from the Gansu Provincial Clinical Testing Center. Cryopreserved bacterial stocks (-80°C) were resuscitated in Trypticase Soy Broth (TSB; Solarbio, Beijing, China) and cultured overnight at 37°C under continuous agitation (200 rpm). The overnight culture was then diluted 1:10,000 in fresh TSB medium and further incubated until reaching the exponential growth phase (4–6 hours).

### Reagents

Commercial reagents were obtained as follows: Annexin V-FITC/PI Cell Apoptosis Detection Kit (Saiweier Biotechnology Co., Ltd., Wuhan, China); BCA Protein Concentration Assay Kit (Yeasen Biotechnology Co., Ltd., Shanghai, China); LIVE/DEAD™ BacLight™ Bacterial Viability Kit (Thermo Fisher Scientific, Waltham, MA, USA); Reactive Oxygen Species Detection Kit (Beyotime Institute of Biotechnology, Shanghai, China).

### Peptide synthesis

The MPX was synthesized by Shanghai Chu Peptide Biotechnology Co., Ltd (Shanghai, China) through solid-phase N-9-fluoromethoxycarbonyl (Fmoc) strategy followed by high-performance liquid chromatography (HPLC) purification, with a certified purity ≥95%. The MPX was dissolved in dimethyl sulfoxide (DMSO) to prepare a 25 mg/mL stock solution and stored at -20°C for experimental use. The mass spectrometry (MS) and HPLC profiles of MPX are provided in **Supplementary Material S1**.

### Antimicrobial susceptibility testing

MIC and MBC were determined using the standardized broth microdilution method following Clinical and Laboratory Standards Institute (CLSI) guidelines (CLSI M07-A11, 2023). Logarithmic-phase MRSA USA300 cultures were adjusted to 5 × 10^6^ CFU/mL in Mueller-Hinton broth (MHB). Antimicrobial agents were serially diluted (1–256 μg/mL) in 200 μL MHB within 96-well plates. Controls included: PC: positive control (MH containing MRSA USA300 without MPX); NC: negative control (MH containing MPX without MRSA USA300); BC: blank control (MH only). After 24-hour incubation at 37°C, bacterial viability was assessed via metabolic reduction of 0.1% resazurin dye (Solarbio Co., Ltd., Beijing, China). The MIC was defined as the lowest concentration preventing detectable metabolic activity (no color change from blue to pink). For MBC determination, aliquots from wells with concentrations ≥ MIC were subcultured on tryptic soy agar (TSA) plates and incubated at 37°C for 24 hours. The MBC was identified as the lowest agent concentration resulting in ≥99.9% reduction in viable colony-forming units (CFU) compared to the initial inoculum.

### Growth curve

According to Shen et al. (2023), a bacterial suspension was prepared using 0.5 McFarland standard and diluted 1:200 in TSB containing 0, 16, 32, or 64 μg/mL MPX. The mixture was incubated at 37°C with continuous shaking (160 rpm). Every 2 hours, 200 μL of the culture was transferred to a 96-well plate (Servicebio, Wuhan, China), and the optical density at 600 nm (OD600) was measured using a microplate reader (RT-6500, Rayto Life and Analytical Sciences Co., Ltd., Shenzhen, China).

### Flow cytometry

To evaluate cell viability and apoptosis, the Annexin V-FITC/PI apoptosis detection kit was utilized to verify cell membrane disruption and bacterial death at different process stages. The kit employs FITC-labeled Annexin V to detect early apoptosis through a mechanism based on phosphatidylserine (PS) translocation – during early apoptosis, PS relocates from the inner to the outer leaflet of the cell membrane, exposing it extracellularly. Annexin V, a 35–36 kDa Ca²^+^-dependent phospholipid-binding protein, specifically recognizes surface-exposed PS with high affinity. Propidium iodide (PI), which cannot penetrate intact membranes, facilitates discrimination between viable early apoptotic cells (Annexin V^+^/PI^-^) and necrotic/late apoptotic cells (Annexin V^+^/PI^+^).

Briefly, log-phase bacteria (1 × 10^6^CFU/mL) were treated with 0, 16, 32, or 64 μg/mL MPX at 37°C with 220 rpm agitation for 6 h, followed by sterile water washing. Bacterial suspensions were stained with 5 μL Annexin V-FITC and 5 μL PI solution, incubated in dark at room temperature for 15 min, then mixed with 400 μL pre-cooled Binding Buffer. Flow cytometry analysis was performed using an EasyCell206A1 flow cytometer (WeiGong Co., Ltd., Shenzhen, China) with standard FITC (Ex/Em 488/530 nm) and PI (Ex/Em 535/617 nm) detection channels.

### Scanning electron microscopy

To investigate the morphological changes induced by MPX on USA300, bacterial suspensions (1.0×10^6^CFU/mL) were treated with MPX at final concentrations of 0, 16, 32, or 64 μg/mL, or with PBS as a control. After 2 hours of incubation at 37°C, the suspensions were centrifuged at 5,000 rpm for 3 minutes at 4°C. The cell pellets were washed three times with PBS and fixed overnight at 4°C with 2.5% glutaraldehyde (Servicebio, Wuhan, China). Subsequently, the bacteria were dehydrated in a graded ethanol series (30%, 50%, 70%, 90%, and 100%). Morphological alterations were visualized using a Hitachi Regulus SU8100 scanning electron microscope (Tokyo, Japan).

### Bacterial viability

The viability of bacterial populations was quantitatively assessed using a dual-fluorescence staining protocol coupled with CLSM(Leica TCS-SP8, Germany) following established methodology (Zapata et al., 2008). Bacterial suspensions (1.0×10^6^CFU/mL) were exposed to MPX at graded concentrations (16, 32, and 64 μg/mL) through co-incubation at 37°C for 60 minutes. Post-treatment samples were centrifuged at 2,000 ×g for 5 min, followed by two sequential PBS washes and resuspension in fresh PBS. Dual fluorescent staining was performed by introducing 10 mM SYTO 9 (viability marker) and 10 mM propidium iodide (PI, membrane integrity indicator) to bacterial aliquots, with subsequent dark incubation at 37°C for 15 min. Residual unbound fluorophores were eliminated through three PBS washing cycles. CLSM imaging was conducted using standardized parameters: 488 nm excitation with 500–550 nm emission collection for SYTO 9, and 543 nm excitation with 600–650 nm detection for PI. Image quantification was performed using Leica LAS X 3D analysis software.

### Membrane fluidity assay

The experimental protocol was executed as follows:Logarithmic-phase bacterial cultures were harvested via centrifugation (5,000 ×g, 10 min) and subjected to dual washing cycles with sterile phosphate-buffered saline (PBS, pH 7.4). The membrane-sensitive fluorophore Laurdan (Sigma-Aldrich, CAS:74515-25-6) was loaded into bacterial suspensions at 10 μM working concentration, with subsequent dark incubation at 37°C for 10 min under aerobic conditions. Post-labeling, cellular pellets were obtained through centrifugation (8,000 ×g, 5 min) followed by triple PBS wash cycles. Aliquots (100 μL) of the labeled suspension were transferred to black-walled, clear-bottom 96-well microplates and combined with 100 μL of MPX solutions prepared at two-fold increasing concentrations (32, 64, 128, 256 μg/mL), 50 mM benzyl alcohol (BA) was used as a positive control. After 60-minute dark incubation at 37°C, fluorescence spectra were captured using a temperature-controlled multimode microplate reader (SpectraMax^®^ Gemini XPS fluorescence microplate reader (Molecular Molecular Devices Co., Ltd., Shanghai, China)) configured with the following optical parameters: excitation λ = 350 nm (5 nm bandwidth), emission λ = 435 nm and 490 nm (10 nm bandwidth). Membrane physicochemical properties were evaluated using Laurdan’s generalized polarization index, computed according to the standardized formula:

The generalized polarization (GP) of Laurdan was calculated using the formula: GP = (I_435_ - I_490_)/(I_435_ + I_490_).

### Reactive oxygen species measurements

The intracellular reactive oxygen species (ROS) generation was monitored using 2’,7’-dichlorodihydrofluorescein diacetate (DCFH-DA) as a fluorescent probe. Bacterial suspensions (1.0 × 10^6^CFU/mL) were treated with MPX at subinhibitory and inhibitory concentrations (0.5×, 1×, and 2× MIC) for 30 min at 37°C, following a modified protocol (Qi et al., 2021). Subsequently, 10 μM DCFH-DA (final concentration) was added to the mixtures and incubated in darkness for 15 min. Cells were then pelleted by centrifugation, washed three times with phosphate-buffered saline (PBS), and resuspended in 1 mL PBS. Fluorescence intensity was quantified using a fluorescence microplate reader with excitation/emission wavelengths set to 485 nm and 528 nm, respectively. Each sample was tested in triplicate.

### Biofilm semiquantitative assay

Overnight cultures of USA300 were prepared and subsequently diluted 1:100 in tryptic soy broth supplemented with 0.5% glucose (TSBG) to achieve a standardized inoculum of 1×10^7^CFU/mL. A two-fold serial dilution of MPX was prepared in TSBG medium, generating concentration gradients from 4 to 68 μg/mL. Following 24 h of static incubation at 37°C under microaerophilic conditions, the planktonic cells were removed by careful aspiration. Adherent biofilms were subsequently washed thrice with phosphate-buffered saline (PBS, pH 7.4) using gentle agitation. Cellular fixation was performed with 200 μL methanol solution for 15 min at ambient temperature. Following methanol removal, the plates were air-dried for 10 min prior to staining with 200 μL of 1% (w/v) crystal violet solution for 8 min. Excess stain was removed by thorough rinsing under deionized water until the effluent became colorless. After complete air-drying, biofilm-incorporated dye was solubilized using 200 μL 30% (v/v) glacial acetic acid solution with 30 min incubation at 37°C. Quantitative analysis was performed by measuring optical density at 600 nm (OD_600_) using a microplate reader. All experimental conditions were assessed in three independent replicates.

### Effect of MPX on the initial adhesion phase of MRSA USA300 biomembranes

We followed a method previously reported by Shen et al. (2023). Log-phase USA300 cultures were inoculated into TSBG containing 0.5% glucose at 1:200 dilution. Aliquots (2 mL) were dispensed into 6-well plates (Servicebio, China) and treated with 16 μg/mL MPX or vehicle control for 3 h at 37°C under aerobic conditions. Following three PBS washes to remove non-adherent cells, adherent bacteria were harvested using sterile cell scrapers (Biosharp, China) in 1 mL PBS. Serially diluted suspensions were spot-plated (10 μL aliquots) on tryptic soy agar (TSA) for colony-forming unit (CFU) enumeration after 24 h incubation.

### MPX against biofilm

To assess the biofilm formation capacity of USA300 and the inhibitory effects of MPX, CLSM was employed to analyze biofilm viability and spatial distribution. Biofilms were cultivated in glass-bottom culture dishes (Biosharp, China). Overnight cultures of MRSA were diluted 1:100 in TSBG (tryptic soy broth with 0.5% glucose), and 2 mL aliquots were dispensed into dishes containing MPX at final concentrations of 16–32 μg/mL. Vehicle-treated controls (0 μg/mL MPX) were included. After 24 h static incubation at 37°C, non-adherent cells were removed by triple PBS washing. Biofilms were dual-stained using SYTO 9 (5 μM) and propidium iodide (PI, 30 μM) from the LIVE/DEAD^®^ BacLight™ Bacterial Viability Kit. Stained samples were incubated in darkness (25°C, 20–30 min) and imaged using a Leica TCS SP8 CLSM system (Leica Microsystems, Germany) with sequential SYTO 9: Ex/Em 488 nm/500–550 nm; PI: Ex/Em 561 nm/600–650 nm.

### Destructive effect of MPX on mature MRSA biofilms

Biofilms were cultivated following established protocols with modifications. After 24 h static incubation at 37°C, planktonic cells were removed by gentle aspiration. Fresh TSBG media containing 64 μg/mL MPX (test group) or vehicle control (MPX-free TSBG, negative control) were prepared under aseptic conditions. Each petri dish received 2 mL of the respective media and underwent secondary incubation for 6 h under identical static conditions. Biofilm viability was assessed using the LIVE/DEAD^®^ BacLight™ Bacterial Viability Kit, with dual staining achieved through 5 μM SYTO 9 and 30 μM propidium iodide (PI) in PBS. Following 20–30 min dark incubation at 25°C, samples were subjected to CLSM analysis using a Leica TCS SP8 system.

### Assessment of MPX cytotoxicity

The cytotoxicity of MPX against rBMSC cells was analyzed using a Cell Counting Kit-8 (CCK-8). To exclude the influence of the solvent on cytotoxicity, 0.1% DMSO (final concentration) was used as the solvent control. rBMSC cells were seeded into 96-well microplates at a density of 1×10^4^ cells per well in 100 μL of culture medium. After incubation for 12 hours at 37°C in a 5% CO_2_ incubator, the cells were treated with complete DMEM medium containing 8–512 μg/mL MPX, 0.1% DMSO (solvent control), or MPX-free complete medium (200 μL per well). The untreated control group was maintained with complete medium without MPX and DMSO. After 24 hours of culture, the supernatant was discarded, and the cells were washed twice with PBS. Subsequently, 100 μL of serum-free DMEM and 10 μL of CCK-8 reagent (total volume: 110 μL per well) were added to each well. The plates were incubated in the dark for 1 hour, and the absorbance was measured at 450 nm using microplate reader (OD_450_).


$$\text{The relative growth rate (\%)}=\frac{A_{450\text{ nm test well}}-A_{450\text{ nm blank control}}}{A_{450\text{ nm negative control}}-A_{450\text{ nm blank control}}}\times 100\%$$

### Transcriptomics analysis sample preparation

To investigate MPX-induced transcriptional responses, MRSA USA300 suspensions (OD600 = 0.6) were treated with subinhibitory MPX (0.5×MIC=16μg/mL) or vehicle control (0.1% DMSO in TSB) for 4 h at 37°C with shaking (180 rpm). Bacterial cells were harvested through centrifugation: Primary separation was performed at 6,000 × g for 5 min (4°C) to remove the supernatant. Final pellets were flash-frozen in liquid nitrogen and stored at -80°C in RNAprotect Bacterial Reagent (Qiagen) until processing. AigenX Biosciences Co., Ltd. (Shanghai, China) processed the samples, with each group run in triplicate.

### RNA library construction and sequencing performance

RNA sequencing (RNA-seq) was performed as follows. Strand-specific libraries were constructed using the Illumina NovaSeq 6000 platform (Illumina, San Diego, CA, USA) Total RNA quality was assessed by measuring concentration and purity with a NanoDrop 2000 spectrophotometer (Thermo Fisher Scientific, USA), while RNA integrity was verified using an Agilent 2100 Bioanalyzer (Agilent Technologies, USA) and agarose gel electrophoresis. Following ribosomal RNA (rRNA) depletion, mRNA was fragmented and reverse-transcribed into double-stranded cDNA. The cDNA underwent end repair, adapter ligation (containing P5/P7 and index sequences), and size selection (370–420 bp) to finalize library preparation. Libraries were rigorously quality-controlled using a Qubit 2.0 Fluorometer (for quantification) and an Agilent 2100 Bioanalyzer (for insert size validation), ensuring an effective library concentration >1.5 nM. Qualified libraries were pooled and subjected to paired-end sequencing on the Illumina platform, utilizing sequencing-by-synthesis (SBS) technology to generate high-quality reads. Differential expression analysis was conducted with a significance threshold of *p* < 0.05.

### RNA-seq data analysis

Following sequencing, raw data were analyzed with the edgeR package to identify differentially expressed genes (DEGs) using the thresholds of |log2 (fold change)| ≥ 1 and *p* < 0.05. Subsequently, the DEGs were subjected to Gene Ontology (GO) functional annotation and Kyoto Encyclopedia of Genes and Genomes (KEGG) pathway enrichment analyses (*p* < 0.05) to uncover biological processes or signaling pathways significantly associated with the identified DEGs.

### RT-qPCR verification

To verify the accuracy of the transcriptome data, seven DEGs involved in different pathways were selected for qRT-PCR analysis and the relative fold changes were calculated after normalization using the comparative CT method. Thermal cycling parameters were as follows: initial denaturation at 95°C for 30 seconds, followed by 40 cycles of 95°C for 15 seconds and 60°C for 10 seconds, and extension at 72°C for 30 seconds. Meanwhile, the expression level of 16S rRNA was defined as an internal control.

### Statistical analysis

All experiments were independently performed in triplicate. Statistical analyses were conducted as follows: comparisons between two groups were analyzed using an unpaired two-tailed Student’s t-test, while comparisons involving more than two groups were evaluated by one-way analysis of variance (ANOVA) followed by Dunnett’s or Tukey’s *post hoc* tests, depending on the experimental design. Statistical significance was defined as p < 0.05, with asterisks indicating specific thresholds: *p < 0.05*, **p < 0.01*, and ***p < 0.001*.

## Results

### MIC and MBC of MPX against MRSA USA 300

**Figure 1:**
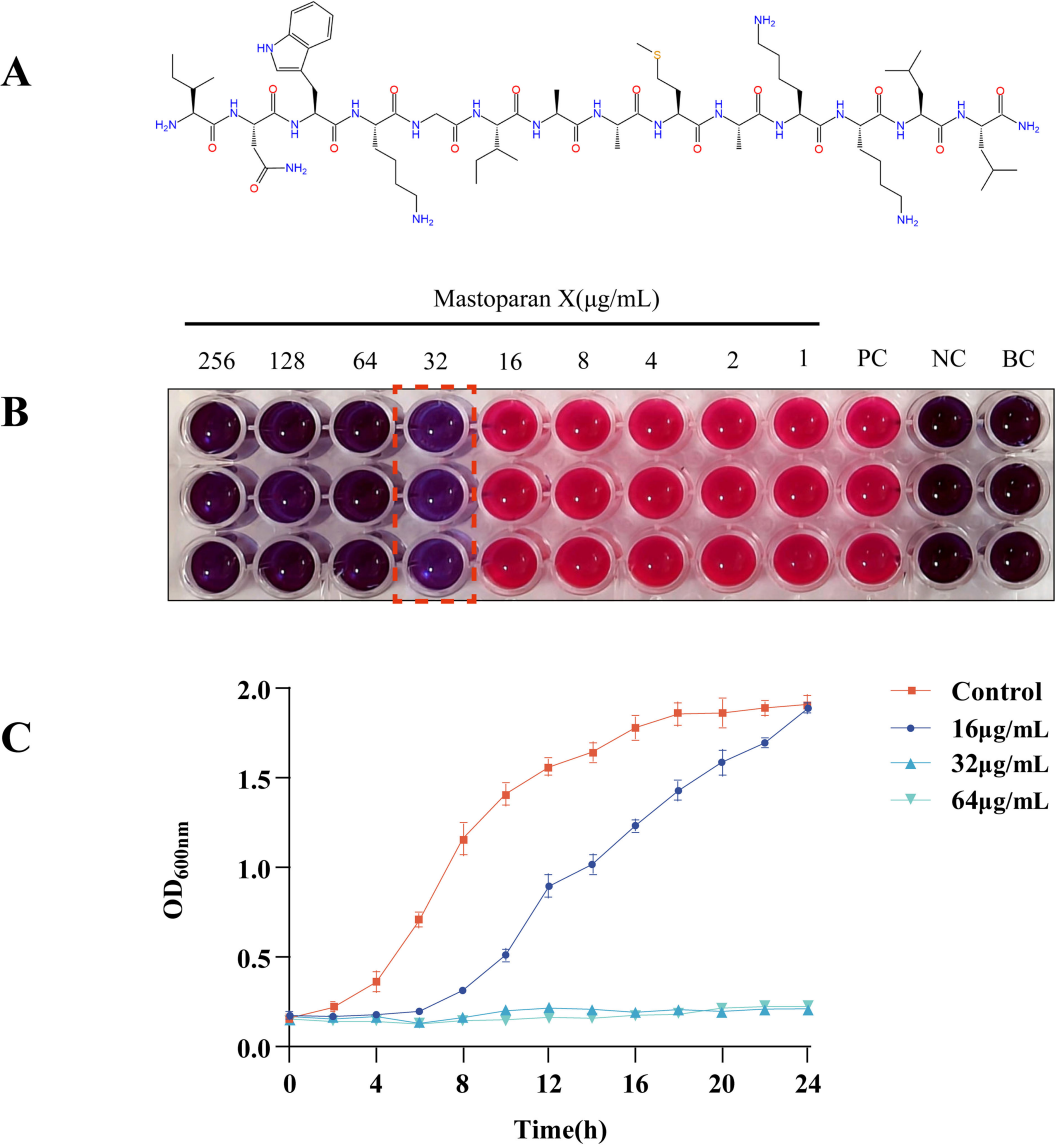
Mastoparan X has antimicrobial activity against MRSA USA300. **(A)** Chemical structure of Mastoparan X; **(B)** Blue indicates inhibited bacterial metabolism (may represent dead cells or live bacteria completely suppressed by the drug), pink reflects metabolically active live bacteria, and the value marked by the red dashed box corresponds to the MIC of Mastoparan X against MRSA USA300 (32 μg/mL). The left well represents 2×MIC (64 μg/mL), while the right well is 0.5×MIC (16 μg/mL); **(C)** Effect of Mastoparan X on bacterial growth.

**Figure 1A** shows the structure of MPX. Antimicrobial susceptibility testing was conducted to further examine the antimicrobial efficacy of MPX against MRSA USA300. MPX exhibited significant antibacterial activity against MRSA, with an MIC of 32 μg/mL (**Figure 1B**). On the other hand, MBC was determined as 64 μg/mL.

### Growth curve

We further tested MPX’s growth inhibitory effect on USA300. Growth was compared between treatments with and without MPX by plotting growth curves. The growth curves showed that at a concentration of 16 μg/mL (**Figure 1C**), MPX prolonged the logarithmic growth period and increased the time required for the bacteria to reach the stationary phase, whereas, according to the data, MPX at concentrations of 32 μg/mL and 64 μg/mL inhibited the growth of MRSA USA300.

### Effect on apoptosis

**Figure 2:**
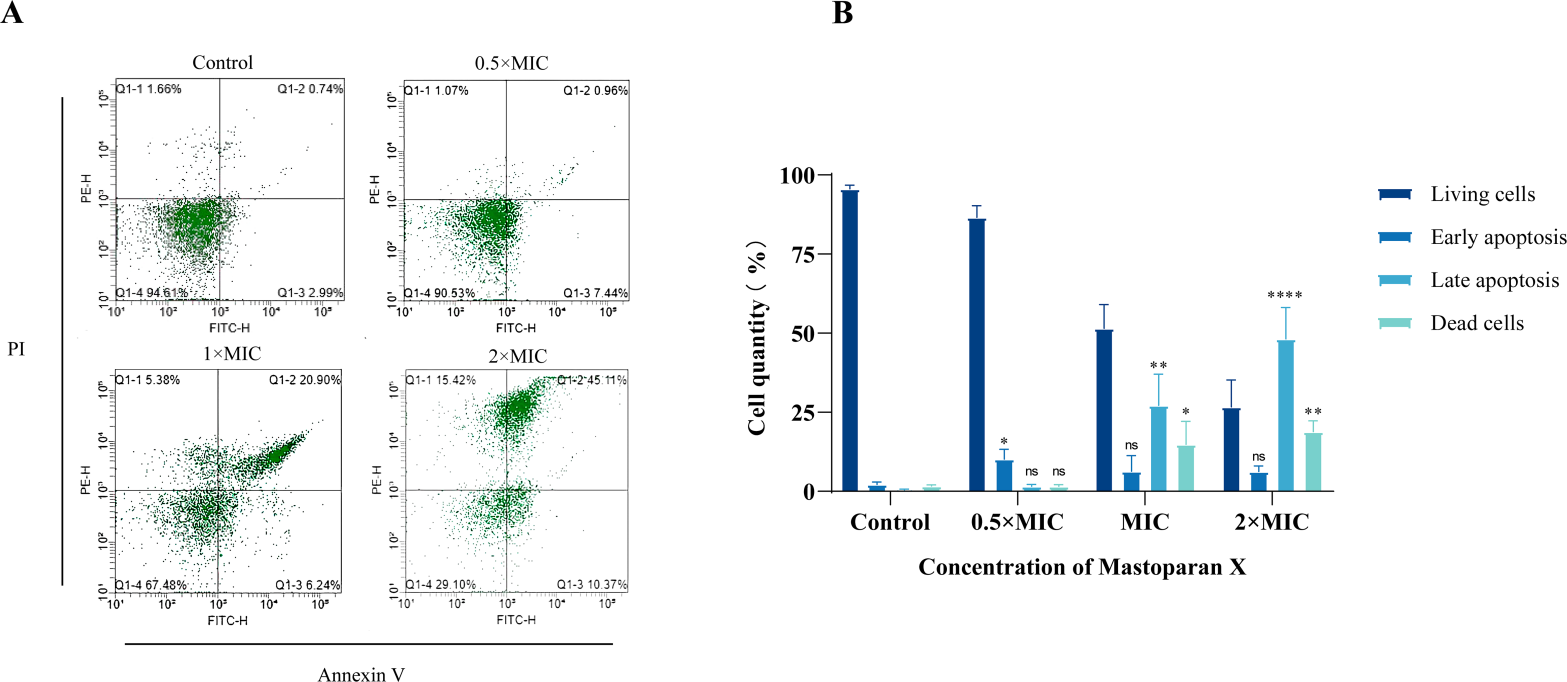
Flow cytometry. **(A)** Cell apoptosis of USA 300 treated with different concentrations of Mastoparan X. **(B)** The apoptosis rate of USA 300 treated with different concentrations of Mastoparan X.

Cell viability and apoptosis were assessed with Annexin V-FITC/PI Apoptosis Detection Kit to confirm cell membrane disruption and bacterial death at different stages of MPX application, and the results are shown in **Figure 2A**. It is well-known that living cells with intact cell membranes do not allow PI to enter the cell and stain DNA, as shown in the Q4 region; therefore, PI is usually used to assess the intactness of cell membranes. **Figure 2B** shows more intuitively the apoptosis rate before and after MPX’s effect on MRSA. After treatment with MPX at 2 × MIC, 1 × MIC, and 0.5 × MIC concentrations, the percentage of viable cells was 26.6%, 51.5%, and 86.60%, significantly lower than that of the control group at 95.6%. These results confirm MPX’s dose-dependent antimicrobial activity against MRSA.

### Effects on MRSA USA 300 morphology

**Figure 3:**
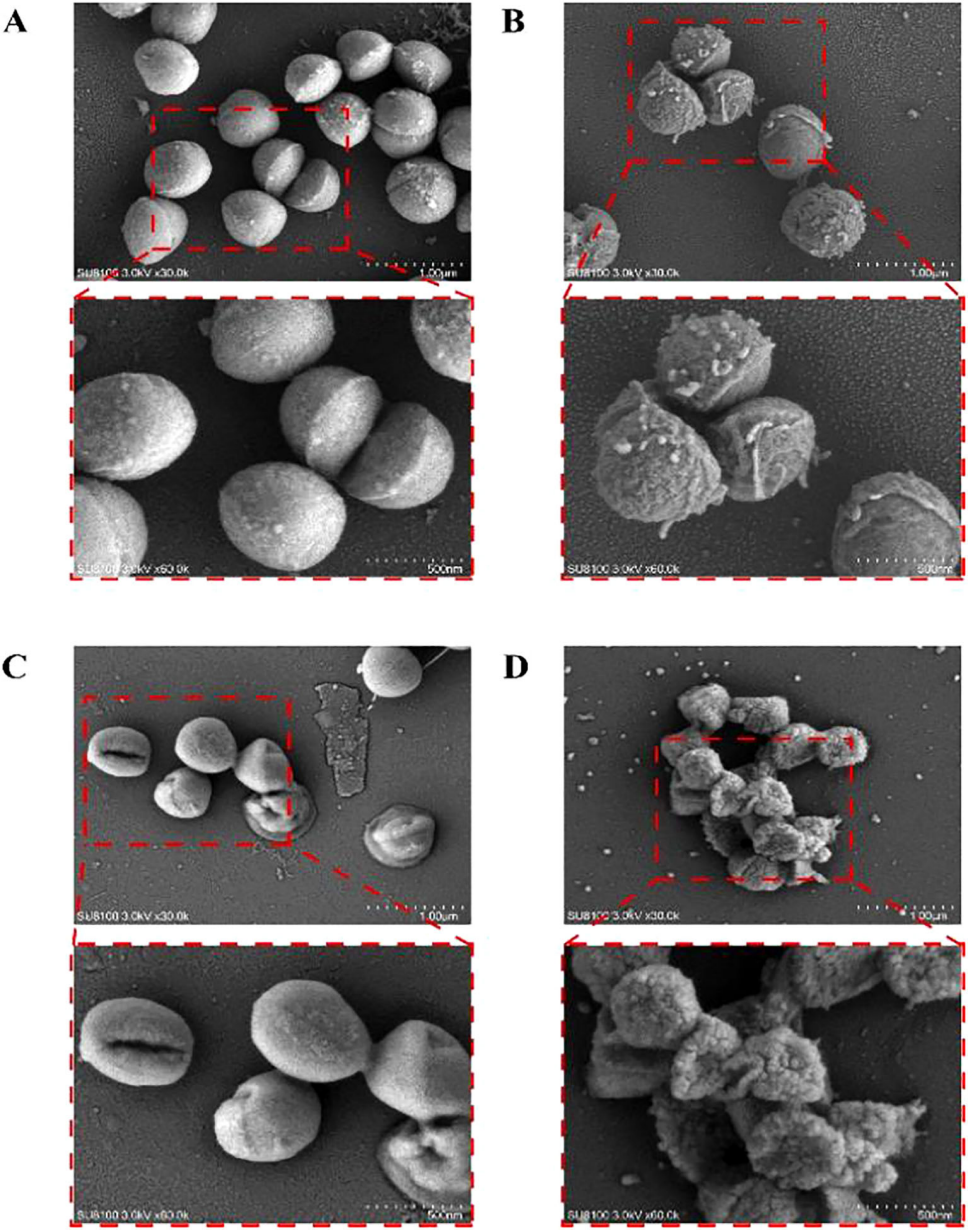
Scanning electron microscopy images of USA 300 exposed and unexposed by Mastoparan X. **(A)** Control. **(B)** 0.5×MIC. **(C)** 1 × MIC. **(D)** 2×MIC.

SEM can directly present the cells’ morphological alterations, with the results illustrated in **Figure 3**. The typical MRSA observed in the figure is spherical, with a smooth, flat surface and intact structure. In contrast, following treatment with varying concentrations of MPX (2 × MIC, 1 × MIC, and 0.5 × MIC), disruption of the USA 300 cells was observed, accompanied by an irregular wrinkling of the surface and the emergence of minute visible pores.

### Effects on membrane integrity

**Figure 4:**
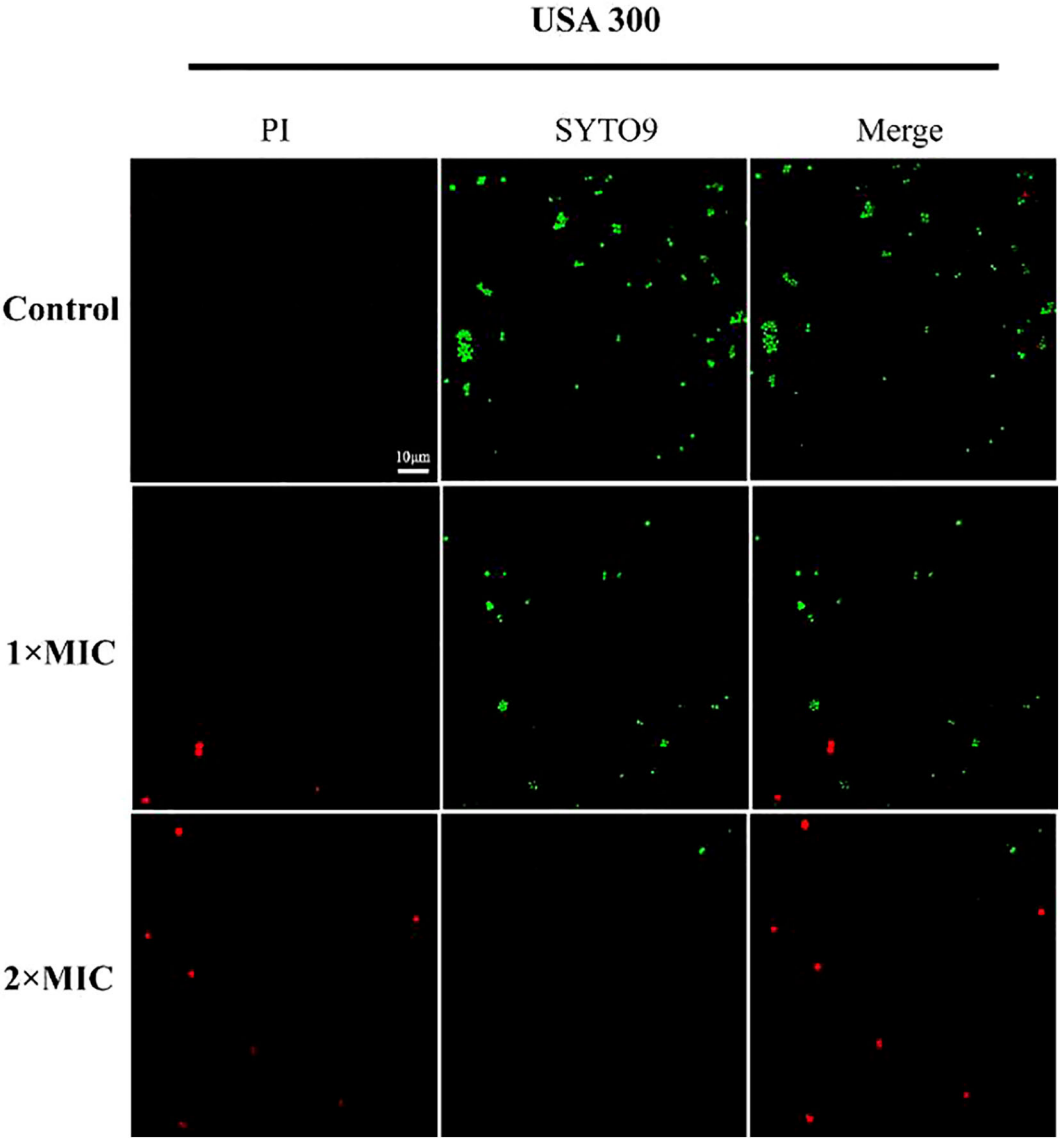
CLSM images of USA 300 exposed and unexposed by Mastoparan X.

In order to ascertain the mode of inhibitory action of MPX, the effect of MPX on the integrity of bacterial cell membranes was determined by the CLSM method, utilizing PI and SYTO9 nucleic acid staining. PI is a red nucleic acid-binding dye that is only capable of penetrating cells with damaged cell membranes, whereas SYTO9 is a green fluorescent nucleic acid staining agent that stains both live and dead bacteria. As illustrated in **Figure 4**, the USA 300 cells in the blank control group were stained with STOY9 but not with PI, indicating that most cells were viable. Conversely, the number and intensity of red fluorescent dots increased concentration-dependently following treatment with MPX at concentrations of 1 and 2 times the MIC.

### Effect on membrane fluidity

**Figure 5:**
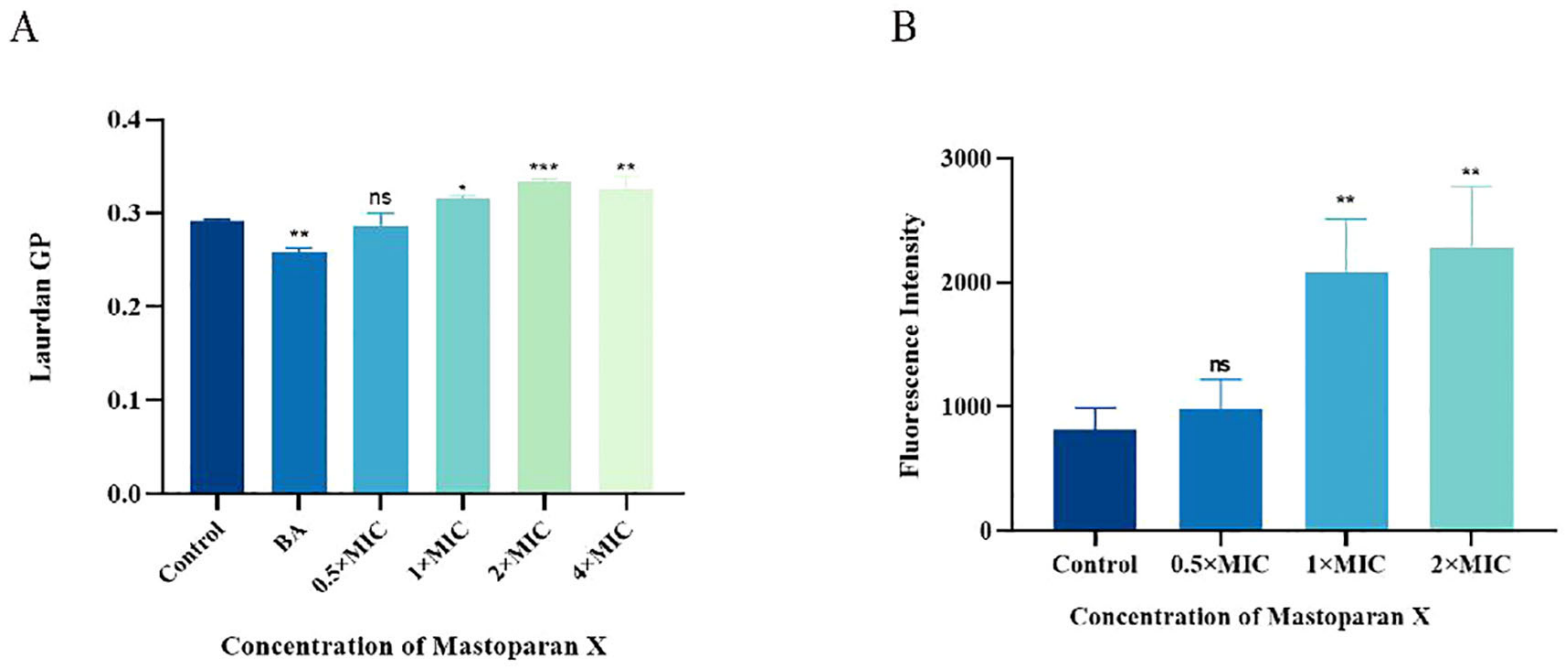
Mastoparan X reduces membrane fluidity and promotes oxidative damage. **(A)** The membrane fluidity of MRSA was evaluated based on Laurdan generalized polarization (Laurdan GP). The benzyl alcohol (50 mM) was used as a positive control. **(B)** Mastoparan X induces the production of ROS in MRSA. Data are expressed as the mean ± SD; n = 3; *p < 0.05, **p < 0.01, *** *p* < 0.001. "ns" (not significant) indicates p ≥ 0.05.

The effect of MPX on the fluidity of *S. aureus* cytoplasmic membranes was assessed by calculating Laurdan GP, a polarity-sensitive fluorescent probe used to detect changes in membrane fluidity. There was an inverse correlation between the Laurdan GP value and the degree of lipid ordering of cytoplasmic membranes; the lower the GP value, the greater the membrane’s fluidity. As shown in **Figure 5A**, The mean Laurdan GP value in the Control group was 0.292, while the BA group showed a mean value of 0.258, significantly lower than that of the Control group. In MPX-treated groups, the mean values of 0.5×MIC, 1×MIC, 2×MIC, and 4×MIC groups were 0.287, 0.316, 0.334, and 0.325, respectively. As MPX concentration increased from 0.5×MIC to 2×MIC, the GP value trended upward (peaking at 2×MIC), indicating a concentration-dependent decrease in cell membrane fluidity, with a slight decline observed at 4×MIC.

### Effect on ROS

Reactive oxygen species (ROS) play an important role in the death of bacteria. ROS fluorescence intensity increased significantly with increasing drug concentration (**Figure 5B**). The mean fluorescence values were: Control (814.19 ± 177.03), 0.5×MIC (979.46 ± 238.85), 1×MIC (2079.1 ± 434.07), and 2×MIC (2284.0 ± 491.35). Statistical analysis revealed significant differences between 1×MIC/2×MIC and Control (*p*<0.001), while 0.5×MIC showed no significant change compared to Control (*p*>0.05). A dose-dependent induction of ROS was observed.

### Effect on biofilm formation

**Figure 6:**
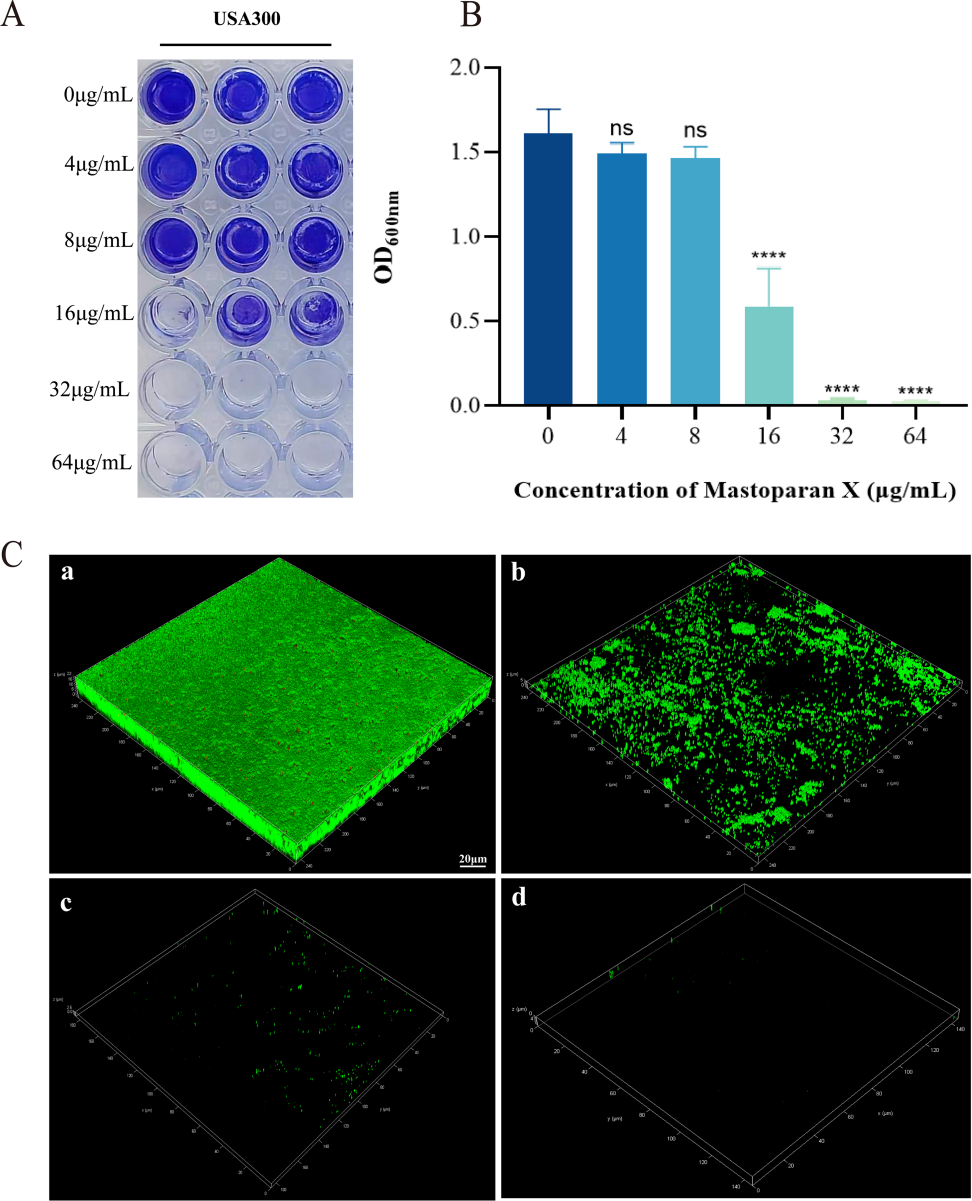
Effect of Mastoparan X on biofilm formation of MRSA USA 300. **(A)** Photographs of crystalline purple-stained microdroplet pores (in triplicate). **(B)** Optical density (OD) values at 600 nm. ****,*P*<0.0001; ns, not significant. **(C)** The biofilms were stained with SYTO 9 and PI: SYTO 9-all cells. a) Control. b) 0.5 × MIC. c) 1 × MIC. d) 2 × MIC. Scale bar = 20 μm.

Forming biofilms and persisters are significant factors associated with chronic implant infections. Crystal violet staining demonstrated that MPX (4–64 μg/mL) exerted a dose-dependent inhibitory effect on USA300 biofilm formation. Significant inhibition (*p* < 0.01) was observed at 16 μg/mL, while higher concentrations (≥32 μg/mL) nearly completely eradicated the biofilm (**Figures 6A, B**). CLSM further confirmed that MPX treatment (16–64 μg/mL) disrupted the three-dimensional architecture of the biofilm, with viable bacterial fluorescence intensity decreasing progressively as the concentration increased (**Figure 6C**).

### Effects on adhesion

**Figure 7:**
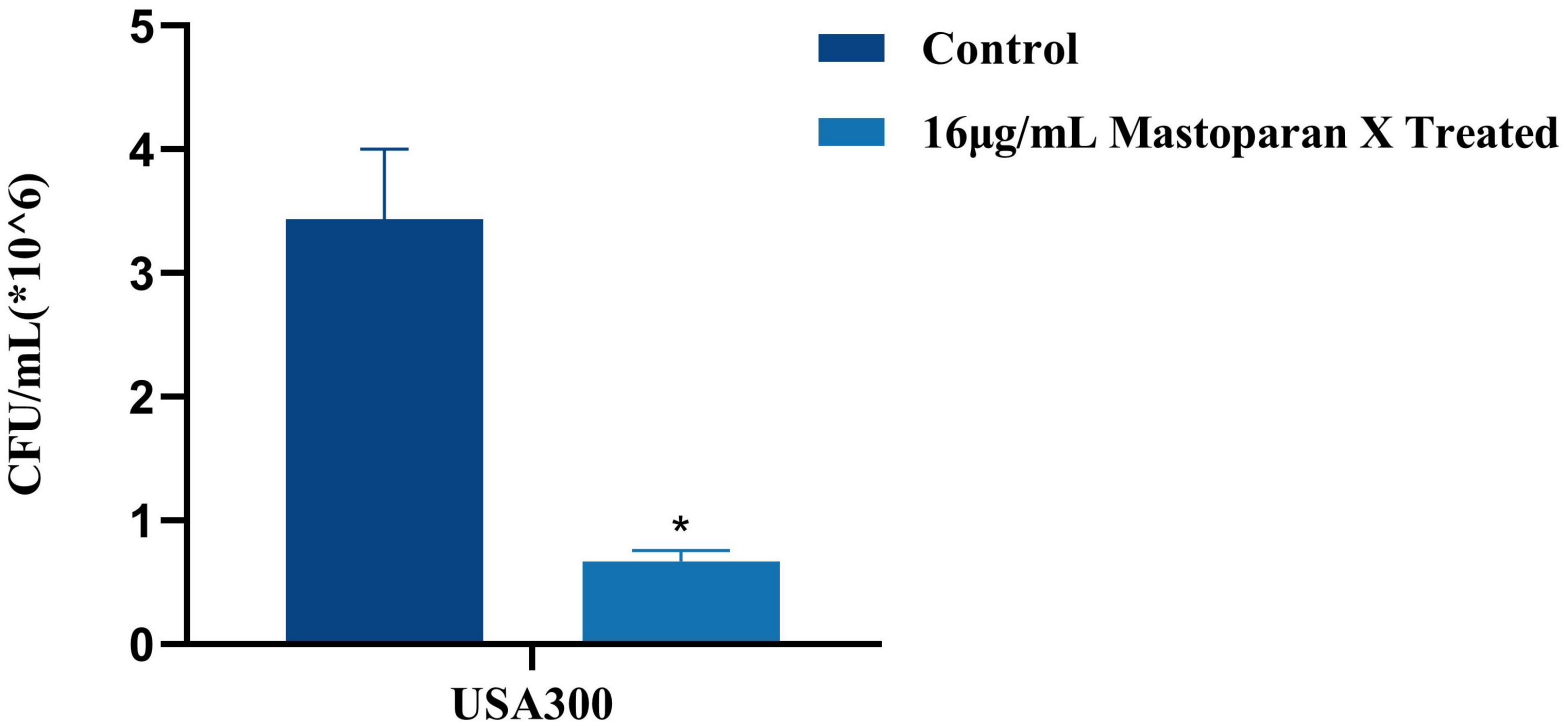
Effect of Mastoparan X on MRSA cell adhesion. The number of attached cells treated with 16μg/mL Mastoparan X or not by colony counting. *P<0.05.

In order to ascertain whether a sub-inhibitory concentration of MPX exerts an inhibitory effect on the adhesion of USA 300, the number of viable bacterial colonies was observed before and after the treatment of MPX by colony counting. In the bacterial adhesion assay, treatment with 16 μg/mL MPX significantly reduced the adhesion of MRSA USA 300 to solid surfaces compared to the control (Control: 3.43 ± 0.55 ×10^6^ CFU/mL; 16 μg/mL MPX: 0.67 ± 0.09 ×10^6^CFU/mL), corresponding to an 80.5% reduction in viable adherent bacteria (**Figure 7**).

### Destructive effects on mature biofilm

**Figure 8:**
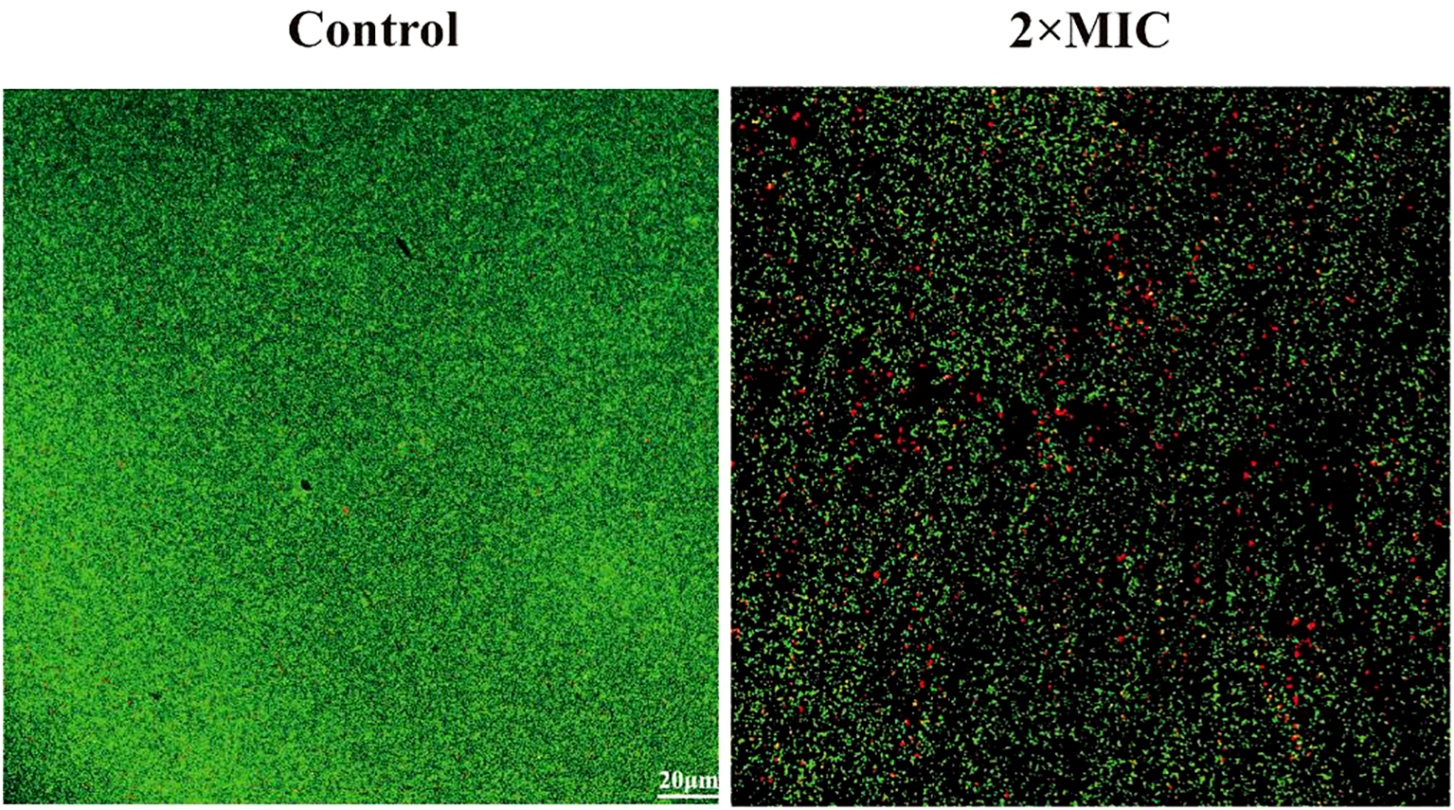
Effect of Mastoparan X on MRSA mature biofilm. Biofilms were treated with Mastoparan X at 2×MIC (64 μg/mL, determined in a prior MIC assay) for 24 h. Control samples received no treatment. Images were captured by confocal microscopy (scale bar: 20 μm).

In order to investigate the destructive effect of MPX on mature biofilms, CLSM was employed to observe the morphology of bacterial death and survival of mature biofilms following a six-hour incubation period with MPX at a 2×MIC. The results, as shown in **Figure 8**, showed that the average green fluorescence brightness of the control group was high, and the bacteria were tightly arranged with each other. In contrast, the biofilm structure of the 2×MIC-treated group became loose, with a decrease in the intensity of green fluorescence and an increase in the intensity of red fluorescence, which indicated that MPX had a destructive effect on mature biofilms.

### Cytotoxicity of MPX

**Figure 9:**
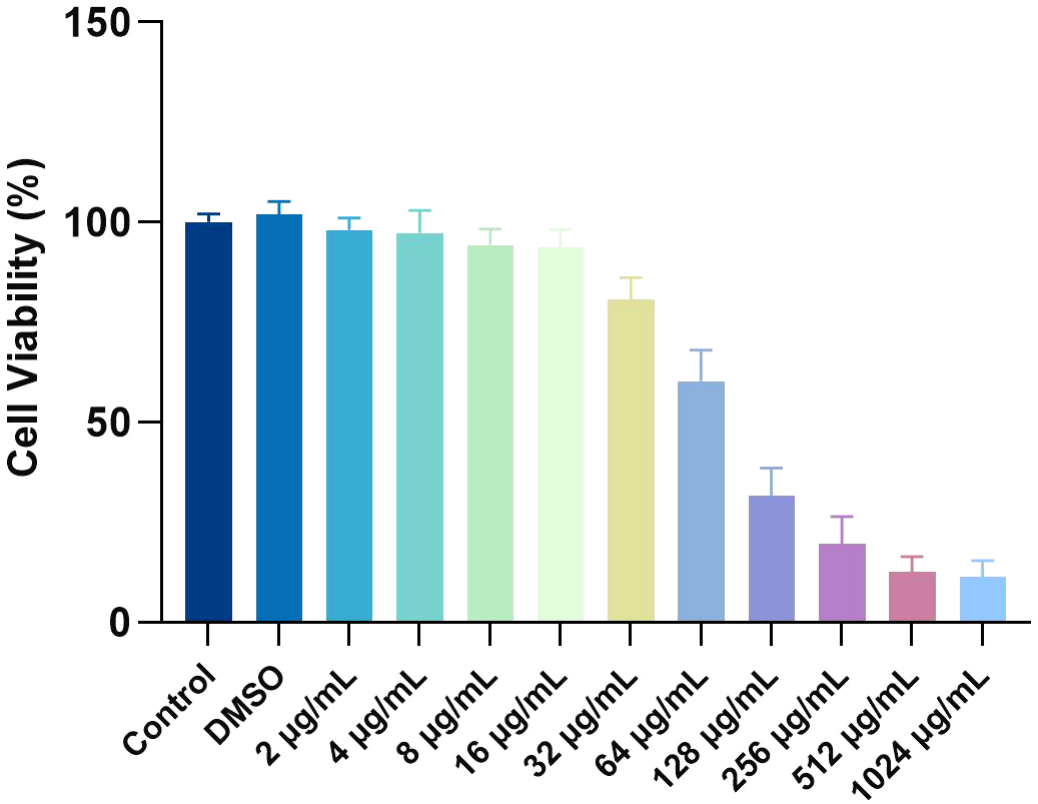
Effect of MPX on rBMSC cells.

The cytotoxicity of MPX toward rBMSCs was evaluated using the CCK-8 assay. Results showed that cell viability in the 0.1% DMSO solvent control group ranged from 98.1% to 101.9%, confirming no solvent-induced toxicity (**Figure 9**). At subinhibitory concentrations (2–16 μg/mL), MPX exhibited minimal cytotoxicity, with cell viability maintained at 80.7–94.3%. However, a dose-dependent cytotoxic effect was observed at higher concentrations: viability decreased to 60.4% at the MIC (32 μg/mL), further declined to 31.6% at the MBC (64 μg/mL, *P* < 0.05), and ultimately dropped to 12.7% at 512 μg/mL. Notably, within the therapeutically effective antimicrobial range (MIC/MBC: 32/64 μg/mL), cell viability remained above 60%, demonstrating that MPX maintains good biocompatibility at bactericidal doses.

### Identification of DEGs

**Figure 10:**
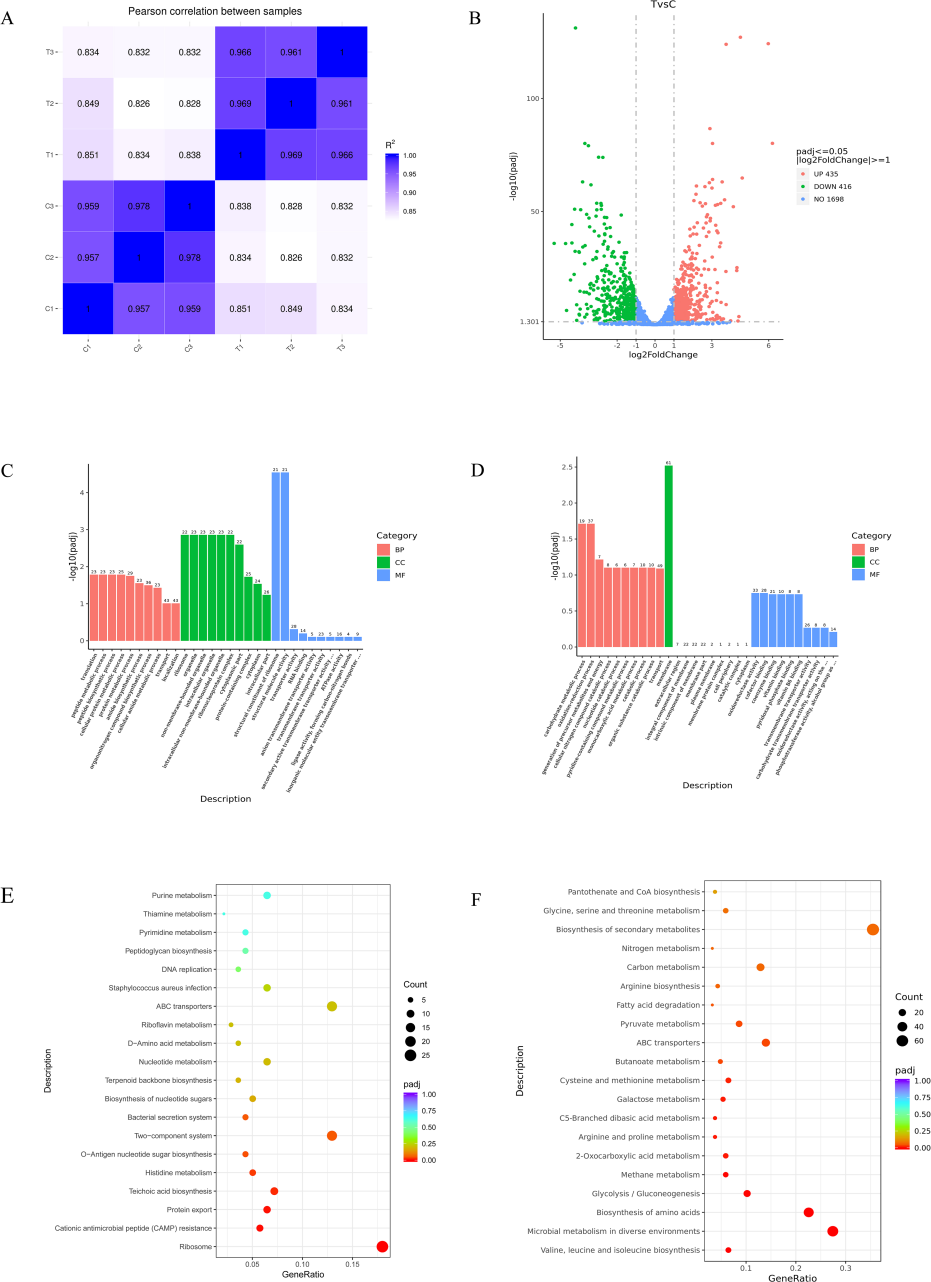
Transcriptome analysis. **(A)** Distribution of gene expression levels; **(B)** Volcano plot of DEGs; **(C)** GO enrichment bar chart for upregulated DEGs; **(D)** GO enrichment bar chart for downregulated DEGs; **(E)** Scatter plot of upregulated KEGG enrichment of DEGs; **(F)** Scatter plot of downregulated KEGG enrichment of DEGs.

To gain further insight into the antibacterial mechanism of MPX against MRSA USA 300, a transcriptome analysis was conducted to assess specific responses at the mRNA level. DEGs were determined for USA 300 in response to MPX treatments at 0.5×MIC (T1, T2 and T3) concentrations and blank controls (C1, C2 and C3). As shown in **Figure 10A**, the correlation analysis of RNA-seq data revealed that Pearson correlation coefficients among samples ranged from 0.83 to 1.00. Intra-group biological replicates (e.g., T1-vs. T2-: 0.969; C3-vs. C2-: 0.978) exhibited significantly higher correlations compared to inter-group pairs (e.g., T1-vs. C1-: 0.851), indicating high consistency among biological replicates and supporting the robustness of experimental design in sample selection. Transcriptomic profiling revealed 851 DEGs in MRSA upon MPX exposure, with 435 significantly upregulated and 416 downregulated (**Figure 10B**). there were 851 differentially expressed genes (DEGs) (435 up-regulated and 416 down-regulated). The most significantly upregulated gene was *sceD* (log_2_FC =6.19). Furthermore, 14 genes exhibited a reduction in expression of greater than fourfold, with the most significantly down-regulated gene being lrgA (log_2_FC = -5.32). **Supplementary Table S2** provides comprehensive data on the DEGs in MRSA between the MPX-treated and control groups. Shows significant differences in transcript levels between control and MPX treated groups.

### GO and KEGG pathway of DEGs enrichment analysis

As shown in **Figure 10C**, the Gene Ontology (GO) enrichment bar chart for upregulated DEGs revealed: significantly enriched biological processes (BP) included translation and protein metabolism, highlighting the critical role of genes in protein synthesis and regulation; Cellular components (CC) were primarily enriched in ribosomes and non-membrane-bound organelles, suggesting significant changes in cellular structure and function; Molecular functions (MF) were dominated by structural components of ribosomes, which may directly affect protein synthesis efficiency. As shown in **Figure 10D**, in the GO enrichment bar chart for downregulated DEGs, BP such as carbohydrate metabolism and oxidation - reduction process are significantly enriched, suggesting that genes play a crucial role in energy metabolism and redox regulation. In terms of CC, genes related to the cell membrane are highly enriched, which may have a significant impact on membrane functions such as cellular material exchange and signal transduction. As shown in **Figure 10E**, in the KEGG enrichment scatter plot for upregulated DEGs, biological pathways such as the ribosome pathway and the cationic antimicrobial peptide (CAMP) resistance pathway are significantly enriched. The ribosome pathway is highly significant with a large number of involved genes. Other pathways are also affected to different degrees. As shown in **Figure 10F**, in the KEGG enrichment scatter plot for downregulated DEGs, in terms of biological processes, multiple pathways such as the biosynthesis of valine and other amino acids and microbial metabolism are significantly enriched. The microbial metabolism pathway involves a large number of genes and may be greatly affected, while pathways like fatty acid degradation involve fewer genes and are relatively less affected.

### RT-qPCR validation

**Figure 11:**
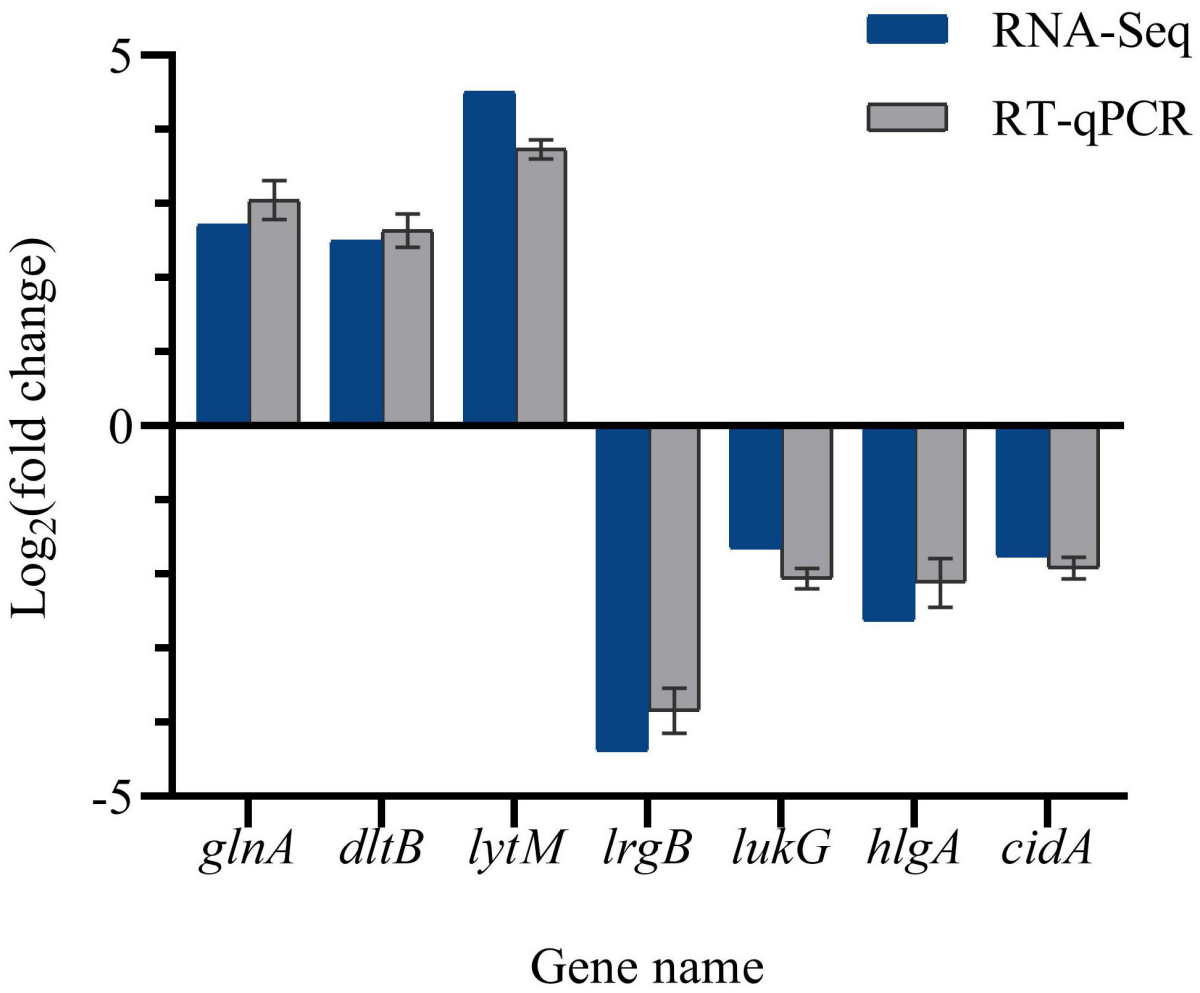
Validation of RNA-seq data by qRT-PCR.

To validate the accuracy of the RNA-Seq data, the expression levels of DEGs were quantified in the same RNA samples using reverse transcription quantitative polymerase chain reaction (RT-qPCR). **Figure 11** illustrates that the expression levels of *lrgB*, *lukG*, *hlgA*, and *cidA* were down-regulated, while the expression levels of *glnA*, *dltB*, and *lytM* were up-regulated. Consequently, the RT-qPCR detection results were consistent with the transcriptome analysis results, thereby confirming the validity of the RNA-Seq data.

## Discussion

The global prevalence of infectious diseases caused by MRSA represents a significant public health concern, given the high morbidity, mortality, and clinical treatment costs associated with these infections. As reported by the Centers for Disease Control and Prevention (CDC), the number of deaths resulting from MRSA infections in the United States exceeds 19,000 annually (Klevens et al., 2007). Consequently, pursuing novel antimicrobial agents to combat MRSA represents a crucial and urgent endeavor. Using AMPs as antibiotic agents represents a novel approach to treating drug-resistant bacteria and biofilms (Lopes et al., 2022). Antimicrobial peptides are a distinctive class of molecules derived from diverse sources exhibiting a broad-spectrum antimicrobial activity.

In this study, we evaluated the *in vitro* antimicrobial activity of MPX against Staphylococcus aureus USA 300. MPX demonstrated potent inhibitory effects, with MIC and MBC values of 32 μg/mL and 64 μg/mL, respectively. Growth curve analysis showed that MPX completely arrested bacterial growth within the first 6 hours, followed by sustained bactericidal activity over 6–24 hours compared to untreated controls. SEM imaging confirmed MPX-induced morphological damage, revealing severe structural alterations in USA 300 cells, including membrane rupture and cytoplasmic leakage. Notably, these membrane-disruptive mechanisms align with prior reports showing that AMPs from *Hermetia illucens* (black soldier fly) larvae kill *S. aureus* via concurrent membrane destabilization and cell cycle inhibition (Liu et al., 2024).

While the exact mechanism of action for AMPs is yet to be elucidated, the prevailing hypothesis suggests their activity is linked to interactions with cell membranes (Li et al., 2022). Many lipopeptide antibiotics, including polymyxin B, daptomycin, bacaosin, and iturin, have been extensively documented to exhibit membrane-disrupting capabilities through such interactions (Maget-Dana and Peypoux, 1994; Seydlová et al., 2019; Zhao et al., 2020).

Microbial cell membranes are important targets for most AMPs. which can exert their broad-spectrum antimicrobial effects by increasing the permeability of cell membranes, inducing the cleavage of cell membranes, and releasing cellular contents (Zhang et al., 2021). Though the SYTO9 and PI mix does not consistently distinguish living from dead cell groups, it is believed to estimate bacterial membrane integrity reasonably. (Berney et al., 2007) The integrity of the cell membrane is important for maintaining the stability of the cell’s internal environment, which is essential for the efficient functioning of metabolic and energy conversion processes. The findings of this study indicate that MPX can disrupt the integrity of the cell membrane in MRSA, suggesting that the cell membrane may be the primary target of MPX’s antimicrobial action.

Apoptosis is a critical metabolic phase in the cell life cycle involving the cessation of cell growth and division, ultimately leading to the orderly death of the cell. In this study, an increase in the concentration of MPX was observed to be positively correlated with the increased rate of bacterial cell apoptosis, a finding consistent with previous research results (Zhang et al., 2017). The PI staining results suggest that MPX may induce apoptosis in MRSA by activating membrane-mediated apoptotic pathways.Apoptosis is a critical metabolic phase in the cell life cycle involving the cessation of cell growth and division, ultimately leading to the orderly death of the cell. In this study, an increase in the concentration of MPX was observed to be positively correlated with the increased rate of bacterial cell apoptosis, a finding consistent with previous research results (Zhang et al., 2017). The PI staining results suggest that MPX may induce apoptosis in MRSA by activating membrane-mediated apoptotic pathways. This mechanism, analogous to the action of other antimicrobial agents, may represent a key pathway for inhibiting *S. aureus* (Tian et al., 2024).

Chemical-lipid interactions can significantly alter membrane fluidity, affecting lipid order, shape, packing, and curvature. (Mykytczuk et al., 2007) Hence, alterations in membrane fluidity serve as a metric for assessing the effects of MPX on membrane lipids. In this study, the membrane fluidity of MRSA was reduced after treatment with MPX. Others (Shireen et al., 2013; Wenzel et al., 2018) have also reported that some AMPs can reduce the membrane fluidity of *S. aureus*. The peptide Hs0_2_ induced an increase in *S. aureus* Lauldan GP values, suggesting that it reduces membrane fluidity (Bessa et al., 2019). Furthermore, it is significant to note that modifications in membrane fluidity augment cellular membrane permeability, potentially leading to the efflux of intracellular constituents and consequentially disrupting multiple cellular processes (Nowotarska et al., 2014).

The accumulation of reactive oxygen species within the cell can damage a number of cellular components, including proteins and lipids, which in turn can affect the generation of ATP. The experimental results revealed that MPX treatment significantly increased the level of ROS in the bacteria, and the resulting oxidative stress damaged MRSA cells. Accordingly, we suggest that MPX can induce bacterial death by disrupting the cell membrane of MRSA and increasing the production of reactive oxygen species.

Biofilm-associated antibiotic resistance poses a significant clinical threat (Gonzalez et al., 2017). The mature biofilm structure functions as a barrier to the inner cells of the bacteria, impeding the penetration of foreign molecules, including numerous small-molecule antimicrobials (Xie et al., 2017). By establishing an *in vitro* model to assess the inhibitory effect of MPX on MRSA biofilm formation, the experimental results indicate that MPX has a significant inhibitory effect on the formation of MRSA biofilms. Its mechanism of action primarily involves disrupting bacterial adhesion to surfaces during the initial attachment phase. Using CLSM to observe the structure of the biofilms, further experimental data confirm that the structure of MRSA biofilms cultured in the presence of MPX is significantly more sparse than those cultured without MPX. This anti-biofilm strategy aligns closely with the mechanism of action of A24, the optimized derivative peptide from AP138, which suppresses early-stage MRSA biofilm formation by targeting the initial attachment phase, while eradicating 40-50% of mature biofilms through membrane-disruptive mechanisms (Zhang et al., 2024).

In contrast to conventional antibiotics that exhibit limited or negligible bactericidal efficacy against biofilm-embedded bacteria due to poor biofilm penetrability (Bhattacharya et al., 2015), certain AMPs demonstrate superior biofilm-penetrating capacity and membrane-disruptive activity, achieving significant bacterial clearance within biofilms and thereby effectively controlling biofilm-associated infections (de Breij et al., 2018; Lakshmaiah Narayana et al., 2020).

In the study of mechanisms of antimicrobial activity, identifying changes in biological function is essential to elucidate their operation. Transcriptome sequencing is a key link between biological genes and functional proteins (Wecke and Mascher, 2011). Today, transcriptome technologies are used to explore the mechanisms of bacterial inhibition in a wide range of substances, including natural products, antibiotics and nanomaterials (Petek et al., 2010).

The bacterial cell membrane, serving as a selectively permeable barrier between the cytoplasm and the extracellular environment, is crucial for maintaining cellular metabolism and energy transduction. Cationic antimicrobial peptides selectively target the negatively charged surface of microbial cell membranes through charge interactions. Their amphiphilic structures can drive the peptides to insert into the phospholipid bilayer of the membrane, induce the formation of membrane pores, and cause the leakage of cytoplasmic contents. Ultimately, they lead to bacterial death by disrupting the membrane integrity (Zhu et al., 2015). It has been reported that AMPs A24, LI14, and L0069 can increase the permeability of the membranes of multidrug-resistant Staphylococcus aureus, cause metabolic disorders, and lead to the rapid death of bacteria (Shi et al., 2022; Zhang et al., 2024). In our investigation, Through GO functional enrichment analysis of CC, it was found that 77% of the DEGs (127/165, *p* < 0.05) were significantly enriched in the functional categories related to the cell membrane. These genes mainly encode membrane - localized proteins (such as transporters and membrane structural proteins), suggesting that the cell membrane function might be the core target of MPX intervention in MRSA. The expression levels of *ProP* and *OpuD* were notably elevated. *ProP* encodes a proline/betaine transporter protein, while *OpuD* encodes a glycine betaine transporter protein. These transporters are believed to enhance the cell’s tolerance to osmotic stress. These findings indicate that MPX can disrupt the cellular membrane of MRSA.

In addition to their involvement in osmotic pressure regulation, amino acids play a pivotal role in the metabolism and growth of microorganisms (Dai et al., 2022) After MPX treatment, several amino acid biosynthesis and catabolic processes of MRSA were affected, and most of the related genes showed down-regulation of transcriptional expression, indicating an imbalanced state of amino acids in MRSA intracellularly.

ABC transporter proteins are structural barriers between the cytoplasm and the external environment and key channels for the exchange of substances between inside and outside the cell, which are important for maintaining the basic life activities of microorganisms (Davidson et al., 2008) The oppA encodes the oligopeptide-binding protein OppA, a subunit of the oligopeptide ABC transporter protein. It recognizes and binds oligopeptides consisting of two to tens of amino acids (Rees et al., 2009) The oppA was down-regulated 1.8-fold after MPX treatment, suggesting that cell membrane recognition of oligopeptides is reduced and that S. aureus has difficulty in replenishing the required amino acids in the body through the uptake of oligopeptides. Staphylococcus aureus can transport antimicrobial peptides via the transmembrane transporter YydJ and the ATP-binding protein YydI. The genes encoding the two proteins, yydJ, and yydI, were significantly down-regulated by 1.94-fold and 2.41-fold, respectively, after MPX treatment.

In this study, the expression of DEGs associated with glycolysis, including adhE, adhP, acsA, gap, and tpiA, as well as those associated with the TCA cycle, such as pckA and sucB, was significantly suppressed following MPX treatment. Inhibition of energy production and stunting biological growth are common intrinsic mechanisms by which bacteriostats act on microorganisms (Sang and Blecha, 2008). MPX treatment disrupts the energy metabolism and transmembrane transport processes in MRSA, leading to a lethal metabolic imbalance. Specifically, MPX inhibits the key enzymes in glycolysis and the TCA cycle, obstructing the production of ATPand depleting the intracellular energy reserves. Meanwhile, the dysfunction of ABC transporters induced by MPX affects the uptake of essential nutrients such as metal ions, sugars, and phosphates. This triggers the compensatory activation of multiple amino acid biosynthesis and metabolic pathways. However, these compensatory responses require a large amount of energy, which exacerbates the energy crisis caused by the inhibition of glycolysis and the TCA cycle. Eventually, the mismatch between energy supply and demand leads to the collapse of bacterial metabolism, and consequently, causes the death of bacteria.

In this study, MPX demonstrated favorable biocompatibility at its effective anti-MRSA concentrations (MIC=32 μg/mL, MBC=64 μg/mL), maintaining >60% viability in rBMSCs. Notably, prior research reported no detectable toxicity in IPEC-J2 cells even at 128 μg/mL MPX, with concurrent anti-inflammatory effects and enhanced intestinal barrier integrity via upregulation of tight junction proteins (Zhao et al., 2021). Furthermore, in both intestinal and testicular barrier models, MPX attenuated inflammation and oxidative stress by suppressing the MAPK pathway, upregulated tight junction proteins (Occludin/Claudin-1), mitigated tissue damage, and inhibited apoptosis, while showing no toxicity to other murine organs (Zhu et al., 2022). These collective findings underscore MPX’s robust biosafety profile at therapeutic doses, providing critical evidence to support its development as a novel antimicrobial agent with balanced efficacy and biocompatibility.

In summary, MPX possessed vigorous anti-MRSA activity with a 32 μg/mL MIC. Our results suggest that the mechanism of anti-MRSA of MPX may involve the disruption of cell membranes, the promotion of apoptosis, and the influence of intracellular metabolic pathways, such as amino acid metabolism, glycolysis, and the TCA cycle. In addition, MPX effectively inhibited the formation of MRSA biofilm and had a destructive effect on mature biofilm. The present study contributes to understanding the mechanism of MPX’s anti-MRSA action and provides a theoretical basis for the study of the antibacterial activity of antimicrobial peptides.

## Data Availability

The original contributions presented in the study are included in the article/**Supplementary Material**. Further inquiries can be directed to the corresponding author.

## References

[B1] Berney M. Hammes F. Bosshard F. Weilenmann H.-U. Egli T. (2007). Assessment and interpretation of bacterial viability by using the LIVE/DEAD BacLight Kit in combination with flow cytometry. Appl. Environ. Microbiol. 73, 3283–3290. doi: 10.1128/AEM.02750-06 17384309 PMC1907116

[B2] Bessa L. J. Manickchand J. R. Eaton P. Leite J. R. S. A. Brand G. D. Gameiro P. (2019). Intragenic Antimicrobial Peptide Hs02 Hampers the Proliferation of Single- and Dual-Species Biofilms of P. aeruginosa and S. aureus: A Promising Agent for Mitigation of Biofilm-Associated Infections. Int. J. Mol. Sci. 20, 3604. doi: 10.3390/ijms20143604 31340580 PMC6678116

[B3] Bhattacharya M. Wozniak D. J. Stoodley P. Hall-Stoodley L. (2015). Prevention and treatment of Staphylococcus aureus biofilms. Expert Rev. Anti Infect. Ther. 13, 1499–1516. doi: 10.1586/14787210.2015.1100533 26646248 PMC5142822

[B4] Costa F. G. Mills K. B. Crosby H. A. Horswill A. R. (2024). The Staphylococcus aureus regulatory program in a human skin-like environment. mBio 15, e0045324. doi: 10.1128/mbio.00453-24 38546267 PMC11077960

[B5] Dai Z. Wu Z. Zhu W. Wu G. (2022). Amino acids in microbial metabolism and function. Adv. Exp. Med. Biol. 1354, 127–143. 34807440 10.1007/978-3-030-85686-1_7

[B6] Davidson A. L. Dassa E. Orelle C. Chen J. (2008). Structure, function, and evolution of bacterial ATP-binding cassette systems. Microbiol. Mol. Biol. Rev. 72, 317–364. doi: 10.1128/MMBR.00031-07 18535149 PMC2415747

[B7] de Breij A. Riool M. Cordfunke R. A. Malanovic N. de Boer L. Koning R. I.. (2018). The antimicrobial peptide SAAP-148 combats drug-resistant bacteria and biofilms. Sci. Transl. Med. 10, eaan4044. doi: 10.1126/scitranslmed.aan4044 29321257

[B8] Gonzalez T. Biagini Myers J. M. Herr A. B. Khurana Hershey G. K. (2017). Staphylococcal biofilms in atopic dermatitis. Curr. Allergy Asthma Rep. 17, 81. doi: 10.1007/s11882-017-0750-x 29063212 PMC6016544

[B9] Guo H. Tong Y. Cheng J. Abbas Z. Li Z. Wang J.. (2022). Biofilm and Small Colony Variants-An Update on Staphylococcus aureus Strategies toward Drug Resistance. Int. J. Mol. Sci. 23, 1241. doi: 10.3390/ijms23031241 35163165 PMC8835882

[B10] Henriksen J. R. Etzerodt T. Gjetting T. Andresen T. L. (2014). Side chain hydrophobicity modulates therapeutic activity and membrane selectivity of antimicrobial peptide mastoparan-X. PLoS One 9, e91007. doi: 10.1371/journal.pone.0091007 24621994 PMC3951324

[B11] Kato H. Hagihara M. Asai N. Shibata Y. Koizumi Y. Yamagishi Y.. (2021). Meta-analysis of vancomycin versus linezolid in pneumonia with proven methicillin-resistant Staphylococcus aureus. J. Glob Antimicrob. Resist. 24, 98–105. doi: 10.1016/j.jgar.2020.12.009 33401013

[B12] Klevens R. M. Morrison M. A. Nadle J. Petit S. Gershman K. Ray S.. (2007). Invasive methicillin-resistant Staphylococcus aureus infections in the United States. JAMA 298, 1763–1771. doi: 10.1001/jama.298.15.1763 17940231

[B13] Koh Jing Jie A. Hussein M. Rao G. G. Li J. Velkov T. (2022). Drug repurposing approaches towards defeating multidrug-resistant gram-negative pathogens: novel polymyxin/non-antibiotic combinations. Pathogens 11, 1420. doi: 10.3390/pathogens11121420 36558754 PMC9781023

[B14] Lakshmaiah Narayana J. Mishra B. Lushnikova T. Wu Q. Chhonker Y. S. Zhang Y.. (2020). Two distinct amphipathic peptide antibiotics with systemic efficacy. Proc. Natl. Acad. Sci. U.S.A. 117, 19446–19454. doi: 10.1073/pnas.2005540117 32723829 PMC7431008

[B15] Lee A. S. de Lencastre H. Garau J. Kluytmans J. Malhotra-Kumar S. Peschel A.. (2018). Methicillin-resistant Staphylococcus aureus. Nat. Rev. Dis. Primers 4, 18033. doi: 10.1038/nrdp.2018.33 29849094

[B16] Li X. Zuo S. Wang B. Zhang K. Wang Y. (2022). Antimicrobial mechanisms and clinical application prospects of antimicrobial peptides. Molecules 27, 2675. doi: 10.3390/molecules27092675 35566025 PMC9104849

[B17] Liu S. Raheel Tariq M. Zhang Q. Wang H. Wang F. Zheng C.. (2024). Dietary influence on growth, physicochemical stability, and antimicrobial mechanisms of antimicrobial peptides in black soldier fly larvae. Insects 15, 872. doi: 10.3390/insects15110872 39590471 PMC11595210

[B18] Lopes B. S. Hanafiah A. Nachimuthu R. Muthupandian S. Md Nesran Z. N. Patil S. (2022). The role of antimicrobial peptides as antimicrobial and antibiofilm agents in tackling the silent pandemic of antimicrobial resistance. Molecules 27, 2995. doi: 10.3390/molecules27092995 35566343 PMC9105241

[B19] Luo Y. Song Y. (2021). Mechanism of antimicrobial peptides: antimicrobial, anti-inflammatory and antibiofilm activities. Int. J. Mol. Sci. 22, 11401. doi: 10.3390/ijms222111401 34768832 PMC8584040

[B20] Maget-Dana R. Peypoux F. (1994). Iturins, a special class of pore-forming lipopeptides: biological and physicochemical properties. Toxicology 87, 151–174. doi: 10.1016/0300-483X(94)90159-7 8160184

[B21] Mykytczuk N. C. S. Trevors J. T. Leduc L. G. Ferroni G. D. (2007). Fluorescence polarization in studies of bacterial cytoplasmic membrane fluidity under environmental stress. Prog. Biophys. Mol. Biol. 95, 60–82. doi: 10.1016/j.pbiomolbio.2007.05.001 17628643

[B22] Nowotarska S. W. Nowotarski K. J. Friedman M. Situ C. (2014). Effect of structure on the interactions between five natural antimicrobial compounds and phospholipids of bacterial cell membrane on model monolayers. Molecules 19, 7497–7515. doi: 10.3390/molecules19067497 24914896 PMC6271777

[B23] Petek M. Baebler S. Kuzman D. Rotter A. Podlesek Z. Gruden K.. (2010). Revealing fosfomycin primary effect on Staphylococcus aureus transcriptome: modulation of cell envelope biosynthesis and phosphoenolpyruvate induced starvation. BMC Microbiol. 10, 159. doi: 10.1186/1471-2180-10-159 20515462 PMC2887449

[B24] Qi M. Zhao R. Liu Q. Yan H. Zhang Y. Wang S.. (2021). Antibacterial activity and mechanism of high voltage electrostatic field (HVEF) against *Staphylococcus aureus* in medium plates and food systems. Food Control 120, 107566. doi: 10.1016/j.foodcont.2020.107566

[B25] Rees D. C. Johnson E. Lewinson O. (2009). ABC transporters: the power to change. Nat. Rev. Mol. Cell Biol. 10, 218–227. doi: 10.1038/nrm2646 19234479 PMC2830722

[B26] Sang Y. Blecha F. (2008). Antimicrobial peptides and bacteriocins: alternatives to traditional antibiotics. Anim. Health Res. Rev. 9, 227–235. doi: 10.1017/S1466252308001497 18983725

[B27] Seydlová G. Sokol A. Lišková P. Konopásek I. Fišer R. (2019). Daptomycin pore formation and stoichiometry depend on membrane potential of target membrane. Antimicrob. Agents Chemother. 63, e01589–e01518. doi: 10.1128/AAC.01589-18 30323037 PMC6325215

[B28] Shen L. Zhang J. Chen Y. Rao L. Wang X. Zhao H.. (2023). Small-molecule compound CY-158–11 inhibits Staphylococcus aureus biofilm formation. Microbiol. Spectr. 11, e0004523. doi: 10.1128/spectrum.00045-23 37166296 PMC10269684

[B29] Shi J. Chen C. Wang D. Wang Z. Liu Y. (2022). The antimicrobial peptide LI14 combats multidrug-resistant bacterial infections. Commun. Biol. 5, 926. doi: 10.1038/s42003-022-03899-4 36071151 PMC9452538

[B30] Shireen T. Singh M. Das T. Mukhopadhyay K. (2013). Differential adaptive responses of Staphylococcus aureus to *in vitro* selection with different antimicrobial peptides. Antimicrob. Agents Chemother. 57, 5134–5137. doi: 10.1128/AAC.00780-13 23856775 PMC3811481

[B31] Tian C Zhao N Yang L. (2024). The antibacterial activity and mechanism of a novel peptide MR-22 against multidrug-resistant Escherichia coli. Front Cell Infect Microbiol. 14, 1334378. doi: 10.3389/fcimb.2024.1334378 38328670 PMC10847306

[B32] Wang Z. Yang Q. Wang X. Li R. Qiao H. Ma P.. (2020). Antibacterial activity of xanthan-oligosaccharide against Staphylococcus aureus via targeting biofilm and cell membrane. Int. J. Biol. Macromol 153, 539–544. doi: 10.1016/j.ijbiomac.2020.03.044 32156542

[B33] Wecke T. Mascher T. (2011). Antibiotic research in the age of omics: from expression profiles to interspecies communication. J. Antimicrob. Chemother. 66, 2689–2704. doi: 10.1093/jac/dkr373 21930574

[B34] Wenzel M. Vischer N. O. E. Strahl H. Hamoen L. W. (2018). Assessing membrane fluidity and visualizing fluid membrane domains in bacteria using fluorescent membrane dyes. Bio Protoc. 8, e3063. doi: 10.21769/BioProtoc.3063 PMC8342135 34532528

[B35] World Health Organization (WHO). (2024). WHO bacterial priority pathogens list, 2024: Bacterial pathogens of public health importance to guide research, development and strategies to prevent and control antimicrobial resistance. Geneva, Switzerland: World Health Organization. Available online at: https://www.who.int/publications/i/item/9789240093461

[B36] Xie T Liao Z Lei H Fang X Wang J Zhong Q. Antibacterial activity of food-grade chitosan against Vibrio parahaemolyticus biofilms. Microb Pathog. 2017 110, 291–297. doi: 10.1016/j.micpath.2017.07.011 28710011

[B37] Zapata R. O. Bramante C. M. de Moraes I. G. Bernardineli N. Gasparoto T. H. Graeff M. S. Z.. (2008). Confocal laser scanning microscopy is appropriate to detect viability of Enterococcus faecalis in infected dentin. J. Endod. 34, 1198–1201. doi: 10.1016/j.joen.2008.07.001 18793919

[B38] Zhang Q.-Y. Yan Z.-B. Meng Y.-M. Hong X.-Y. Shao G. Ma J.-J.. (2021). Antimicrobial peptides: mechanism of action, activity and clinical potential. Mil Med. Res. 8, 48. doi: 10.1186/s40779-021-00343-2 34496967 PMC8425997

[B39] Zhang K. Yang N. Mao R. Hao Y. Teng D. Wang J. (2024). An amphipathic peptide combats multidrug-resistant Staphylococcus aureus and biofilms. Commun. Biol. 7, 1582. doi: 10.1038/s42003-024-07216-z 39604611 PMC11603143

[B40] Zhang L.-L. Zhang L.-F. Hu Q.-P. Hao D.-L. Xu J.-G. (2017). Chemical composition, antibacterial activity of *Cyperus rotundus* rhizomes essential oil against *Staphylococcus aureus* via membrane disruption and apoptosis pathway. Food Control 80, 290–296. doi: 10.1016/j.foodcont.2017.05.016

[B41] Zhao X. Li Z. Kuipers O. P. (2020). Mimicry of a non-ribosomally produced antimicrobial, brevicidine, by ribosomal synthesis and post-translational modification. Cell Chem. Biol. 27, 1262–1271.e4. doi: 10.1016/j.chembiol.2020.07.005 32707039

[B42] Zhao X. Wang L. Zhu C. Xia X. Zhang S. Wang Y.. (2021). The antimicrobial peptide mastoparan X protects against enterohemorrhagic Escherichia coli O157:H7 infection, inhibits inflammation, and enhances the intestinal epithelial barrier. Front. Microbiol. 12, 644887. doi: 10.3389/fmicb.2021.644887 34177825 PMC8222680

[B43] Zhu C. L. Wang L. Zhao X. Q. Yang R. Zhang B. Y. Zhao Y. Y. (2022). Antimicrobial peptide MPX attenuates LPS-induced inflammatory response and blood-testis barrier dysfunction in Sertoli cells. Theriogenology 189, 301–312. doi: 10.1016/j.theriogenology.2022.07.001 35842953

[B44] Zhu X. Zhang L. Wang J. Ma Z. Xu W. Li J.. (2015). Characterization of antimicrobial activity and mechanisms of low amphipathic peptides with different α-helical propensity. Acta Biomater 18, 155–167. doi: 10.1016/j.actbio.2015.02.023 25735802

