# Supplementary material for: Studies on the antibacterial activity of the antimicrobial peptide Mastoparan X against methicillin-resistant Staphylococcus aureus
Source: Front Cell Infect Microbiol. 2025 May 29;15:1552872. doi: 10.3389/fcimb.2025.1552872 (PMC12159006; doi:10.3389/fcimb.2025.1552872)
Supplement: Supplementary file 1 [file Table1.docx]

Mass Spectrometry Report

|   [M+3H]3+  [M+H]+  [M+H]+  [M+2H]2+ | | | | | | | |
| --- | --- | --- | --- | --- | --- | --- | --- |
|  | Sample Description | | Instrument | SHIMADZU LCMS-2020 | | | |
|  | Analyzed date: | 2023/5/16 | Probe: | ESI | | Probe Bias: | ＋4.5kv |
|  | Analyst: | Apeptide | Nebulizer Gas Flow: | 1.5L/min | | Detector: | 1.2kv |
|  | Sample: | IL-14-NH2 | CDL: | -20.0v | | T. Flow: | 0.2ml/min |
|  | M.W.: | 1555.97 | CDL Temp.: | 250 | ^o^C | B. Conc.: | 50%H2O/50%ACN |
|  | Lot. No.: | Apep-1014589 | Block Temp.: | 400 | ^o^C |  |  |

HPLC REPORT

| Sample: | IL-14-NH2  INWKGIAAMAKKLL-NH2 | | |  |
| --- | --- | --- | --- | --- |
| Analyst: | Apeptide | | | |
| Lot No.: | Apep-1014589 | | | |
| Column: | Symmetrix ODS-R, 4.6*250mm, 5μm | | | |
| Solvent A | A: 0.1% Trifluoroacetic Acid in 100% Acetonitrile | | | |
| Solvent B | B: 0.1% Trifluoroacetic Acid in 100% Water | | | |
| Gradient : |  | A | B |  |
|  | 0.0min | 35% | 65% |  |
|  | 25.0min | 60% | 40% |  |
|  | 25.1min | 100% | 0% |  |
|  | 30.0min | Stop | |  |
| Volume: | 10μl | | |  |
| Wavelength: | 220nm | | |  |
| Flow rate: | 1.0ml/min | | |  |

────────────────────────

Rank Time Conc. Area Height

────────────────────────

1 8.537 0.8768 53043 7921

2 8.730 98.27 5944782 514545

3 9.013 0.5718 34595 13493

4 9.788 0.2844 17209 3156

────────────────────────

Total 100
